# Size-resolved spatial distribution analysis of aerosols with or without the utilization of a novel aerosol containment device in dental settings

**DOI:** 10.1063/5.0056229

**Published:** 2021-08-02

**Authors:** Fernando Luis Esteban Florez, Tyler Thibodeau, Toluwanimi Oni, Evan Floyd, Sharukh S. Khajotia, Changjie Cai

**Affiliations:** 1 Department of Restorative Sciences, Division of Dental Biomaterials, College of Dentistry, The University of Oklahoma Health Sciences Center, 1201 N. Stonewall Avenue, Oklahoma City, Oklahoma 73117, USA; 2 School of Industrial and Systems Engineering, The University of Oklahoma, 202 W. Boyd Street, Norman, Oklahoma 73019, USA; 3 Department of Occupational and Environmental Health, Hudson College of Public Health, The University of Oklahoma Health Sciences Center, 801 N.E. 13th Street, Oklahoma City, Oklahoma 73126, USA

## Abstract

The coronavirus disease 2019 pandemic has imposed unprecedented occupational challenges for healthcare professionals. In dentistry, handheld instruments such as air and electric handpieces, ultrasonic scalers, and air/water syringes are capable of generating aerosols, droplets, and splatter, thereby exposing dental professionals to airborne contaminants such as viruses, bacteria, and fungi. The objective of the present study was to determine the spatial distribution of aerosols by size (0.30 to 20.00 *μ*m) and the efficacy of a novel aerosol containment device (ACD) in a large operatory room with 12 dental chairs. Real-time portable laser aerosol spectrometers were used to measure the size-resolved number concentration of aerosols generated by a collision nebulizer. Results reported demonstrate that aerosol number concentrations significantly decreased as a function of distance with or without the utilization of the ACD. The ACD was able to efficiently decrease (up to 8.56-fold) the number and size distribution of particles in a large dental clinic. The novel device demonstrated higher efficiency for particles shown to contain the highest levels of severe acute respiratory syndrome coronavirus 2 in Chinese hospitals, thereby showing great promise to potentially decrease the spreading of nosocomial pathogens in dental settings.

## I. INTRODUCTION

In December of 2019, Chinese health authorities reported on a new type of zoonotic infectious respiratory disease.^1^ The pathogen was soon thereafter identified as SARS-CoV-2 (severe acute respiratory syndrome coronavirus 2), and the resulting infection was named coronavirus disease 2019 (COVID-19).^2^ Coronaviruses such as SARS-CoV (severe acute respiratory syndrome), MERS-CoV (Middle East respiratory syndrome), and SARS-CoV-2^3^ cause a broad range of diseases in birds and mammals.^4^ In humans, coronaviruses are known to cause potentially lethal (mortality rates: 2% to 15%)^5^ pneumonia-like infectious diseases that are clinically translated into patients displaying flu-like symptoms including headache, fever, cough, myalgia, fatigue, sputum production, hemoptysis, and diarrhea.^3^ The common routes for SARS-CoV-2 spread include (i) airborne transmission through inhalation of droplets and aerosols and (ii) direct contact transmission from the exposure of conjunctival, nasal, or oral mucosae to contaminated body fluids.^6–8^

Even though droplet nuclei (1–5 *μ*m), aerosols (<50 *μ*m), droplets, and splatter (>50 *μ*m) have been commonly used to describe gas-based colloidal suspensions containing dispersed particles [either liquid (water, saliva, blood, sputum) or solid (bacteria, virus, fungus, and dental plaque)],^9–11^ recent reports have indicated an urgent need to harmonize discussions about modes of virus transmission across disciplines by clarifying the terminology used to distinguish between aerosols and droplets. According to these studies, a threshold of 100 *μ*m should be used because this size more effectively separates particles' aerodynamic behavior,^12–14^ and their ability to inhale can be used to determine the utility of engineering controls and strategies while providing clear guidance to the public.^12^

Aerosols can be generated by humans, animals, and different types of instruments (e.g., rotary, oscillating, vibrating, piezosurgical, nebulizing).^15^ According to previous studies, particle sizes are typically associated with the process by which they have been generated (e.g., combustion, mechanical, or biological) and significantly influence their aerodynamic behavior (e.g., settling, impaction, and coagulation). In healthcare facilities, nosocomial spreading of pathogens may occur when patients generate respirable particles (diameters <4 *μ*m) by speaking,^16^ sneezing, and coughing, or when pathogens become re-aerosolized after being deposited on surfaces.^17^

In dentistry, the utilization of rotary instruments (low- and high-speed handpieces), ultrasonic scalers, and air–water syringes results in the formation of bioaerosols containing a broad variety of microorganisms^1,15,18,19^ and viruses.^15^ According to a recent study, approximately 78% of dental professionals (n = 700) around the world perceive dental settings as very high risk for the transmission of SARS-CoV-2.^20^ In fact, a previous study indicated that the potential for contamination through an aerosol depends on the amount and quality of saliva, nasal, and throat secretions, and the presence of blood, dental plaque (biofilms), or oral infections (e.g., caries, periodontitis, abscesses).^21^ Other studies have demonstrated that SARS-CoV-2 viral load in the oronasal pharynx tends to vary between 10^2^ and 10^11^ copies/ml of respiratory fluid and the highest concentrations detected were typically found at the onset of COVID-19 symptoms.^22–24^

Kobza *et al.*,^25^ when investigating contamination risks from bioaerosols to dental professionals, have shown that bacterial exposures varied from 1.86 × 10^5^ to 4.3 × 10^5^ bacteria/m^3^ depending on the type of procedure conducted [cavity preparation (24–105 CFU/m^3^) > ultrasonic scaling (42–71 CFU/m^3^) > oral examination (24–62 CFU/m^3^)]. Results reported^25^ indicate that bacteria (17 species, ten sub-types; most prevalent: *Staphylococci* and *Bacilli*) and fungi (seven species, four sub-types; most prevalent: *Penicillium* and *Cladosporium*) were the predominant types of micro-organisms found in dental bioaerosols. Another study demonstrated that ultrasonic scalers were capable of generating splatter even without the utilization of coolant water,^26^ and airborne material spread over a distance of at least 18 in. from the operatory site.^10,26^ Despite these results, a recent study investigating the spatial distribution of contaminants (fluorescein tracer, distances up to 110 cm) promoted by either air-polishing or ultrasonic scaling procedures indicated that higher contamination levels of nearby structures were observed after the execution of air-polishing procedures.^27^ According to the U.S. National Institute for Occupational Safety and Health (NIOSH),^28^ particles with dimensions similar to those found to contain peak levels of SARS-CoV-2 (between 0.25 and 0.50 *μ*m)^29^ may remain suspended in the air (either still or turbulent) for as long as 41 h, which favors the ability of particles of that size range to transmit infectious respiratory diseases like COVID-19, tuberculosis, influenza, and others.^28^ *In vitro* studies investigating the stability and persistence of SARS-CoV-2 in aerosols and on surfaces (plastic, stainless steel, copper, and cardboard) have indicated, through the utilization of a Bayesian regression model, that SARS-CoV-2 can remain infectious while airborne for extended periods of time.^30,31^

In this critical scenario, several attempts to reduce the exposure of dental professionals to bioaerosols with pathogenic potential have been previously reported. These strategies include pre-procedural microbial control using antimicrobial photodynamic therapy,^32^ mouthwash rinse solutions (0.12% chlorhexidine gluconate, cetylpyridinium chloride),^33–36^ high-volume evacuators,^37,38^ in-service instrumentation coolant agents,^39^ and antiseptic agents dispensed directly into the dental unit waterlines (DUWLs).^40^ Since 1996, the Council on Scientific Affairs and the Council on Dental Practice of the American Dental Association (ADA) have recommended^41^ that aerosols be controlled by the utilization of personal protective equipment (PPE), rubber dams, and appropriate positioning of patients.^42^ According to a recent systematic review of the literature, pre-procedural mouth rinse with tempered chlorhexidine (47 °C) is the most effective strategy to reduce aerosol-related bacterial load in dental operatories.^42^

The context presented illustrates the critical need for the characterization of aerosols in dental settings (physical, chemical, and biological properties) and for the development of engineering controls capable of mitigating the exposure of patients and dental professionals to bioaerosols with pathogenic potential. Several methods are available for measuring particulate mass concentrations including filtration, beta gauges, gravimetric mass, tapered element oscillating microbalance, condensation particle counter, differential and scanning mobility analyzers, optical counters, aerodynamic particle sizers, and aerosol mass spectrometers.^43^ Among the quantification approaches cited, aerodynamic particle sizing is preferred over light scattering methods because it measures particle sizes based on their aerodynamic behavior and has a fast response time and sufficient resolution to screen particles with dimensions in the most important range (0.3–20.0 *μ*m) for the spreading of SARS-CoV-2 in healthcare settings.^43^ Despite recent developments in the field of aerosol measurement and control, the ability to accurately determine the spatial spread of aerosols with respect to particle size under controlled experimental conditions is still limited.^44^

In addition, epidemic modeling studies aiming to assess the spread of respiratory diseases in community settings have indicated that it is impossible to specify geometries, ventilation efficiency, and the location of infectious sources (e.g., index patient) in each microenvironment.^45^ Typically, these studies model the risk of infection due to airborne transmission using the Wells–Riley formulation^46,47^ modified by Gammaitoni and Nucci.^48^ When these types of models are applied, five assumptions must be made:^49^ (i) there is only one infectious individual who emits SARS-CoV-2 quanta (quanta = the viral dose in the aerosol required to cause infection)^50,51^ at a constant emission rate (*E* = 970 h^−1^) throughout the event; (ii) there is no prior source of quanta in the space; (iii) the latent period of the disease is longer than the timescale of the model; (iv) the infectious respiratory aerosol quickly becomes discrete (evenly dispersed) throughout the room; and (v) infectious quanta are removed by ventilation, filtration, deposition, or inactivation.^49^ Typical quanta emission rates for respiratory diseases such as measles (5580 q h^−1^)^52^ and tuberculosis (between 1.25 and 30 840 q h^−1^)^53^ have been previously reported to be significantly higher than the quanta reported for SARS-CoV-2 (between 10.5 and 1030 q h^−1^).^54^

Therefore, the objective of the present study was to determine the spatial distribution of aerosols by size (0.30 to 20.00 *μ*m) and the efficacy of a novel aerosol containment device (ACD) using a portable real-time laser aerosol spectrometer in a large operatory room with 12 dental chairs. The novel ACD proposed is expected to potentially control bioaerosols in dental settings, as recommended by the American Dental Association (ADA),^41^ the U.S. Centers for Disease Control and Prevention (CDC), and as regulated by the U.S. Occupational Safety and Health Administration (OSHA).^55^

## II. METHODS AND MATERIALS

### A. Fluorescent microspheres

Fluorescent microspheres (FluoSpheres, Life Technologies Corp., USA) that are carboxylate-modified [diameters = 0.10 (orange), 0.20 (yellow–green), 0.50 (red), 1.00 *μ*m (blue)] or made of polystyrene [diameters = 10.00 (blue–green), and 15.00 *μ*m (blue)] were suspended (2%, v/v) into de-ionized water (DIW) (LAL Reagent Water, Lonza, Switzerland) containing 2% sodium chloride (NaCl, Sigma-Aldrich, USA) in preparation for nebulization procedures.

### B. Aerosol generation and size-resolved spatial distribution analysis

**FIG. 1.**
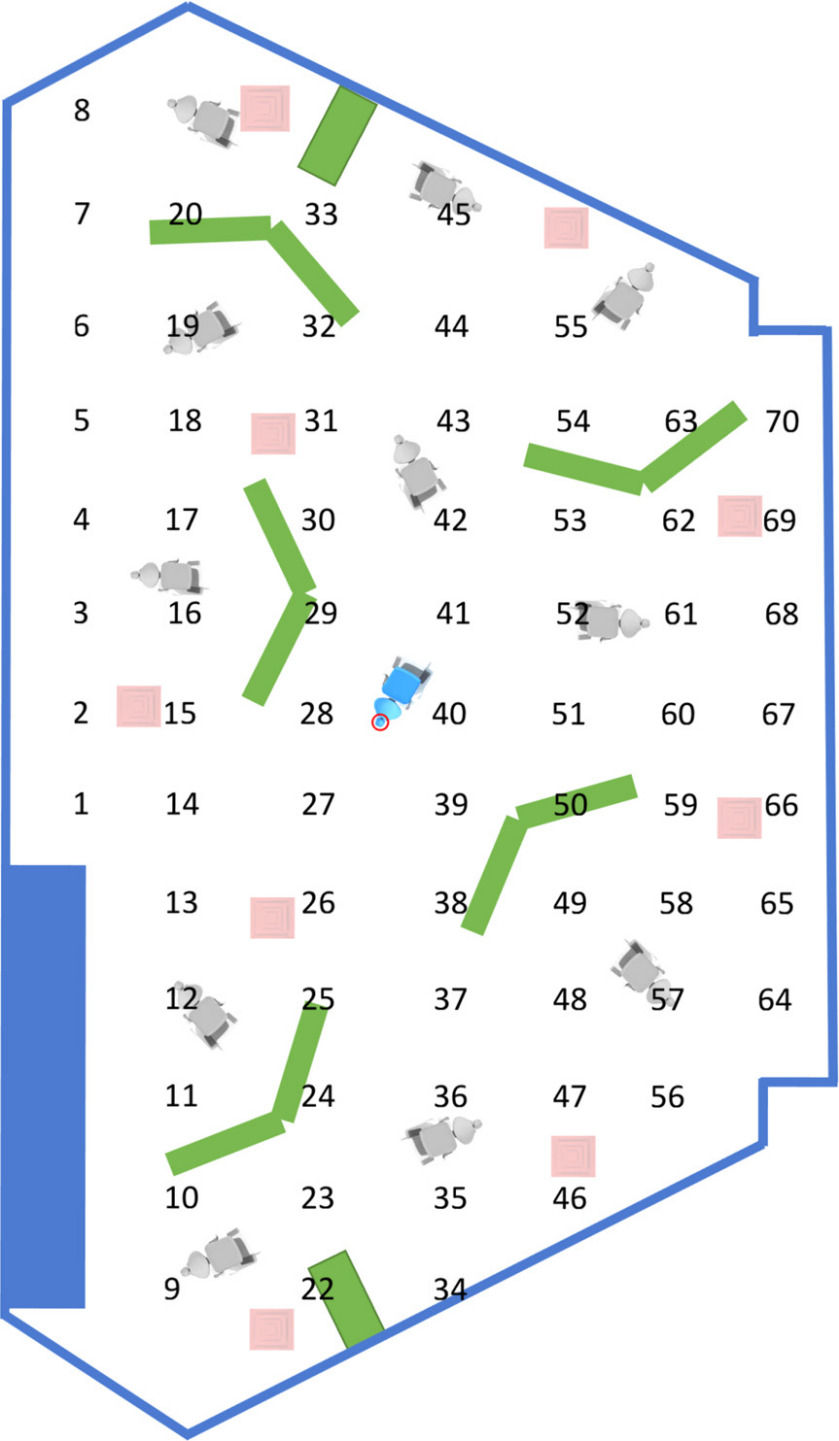
2D model of the Green Clinic illustrating the position of air vents/exhausts (red squares), dental chairs (blue/gray), cabinetry (green), and office space (blue rectangle). The red circle located on the blue dental chair indicates the location of the Collison nebulizer as shown in Fig. 3(a). Numbers (1–70) indicate the locations where spatial distribution measurements took place.

A portable Collison nebulizer [three jet, CH Technologies, Westwood, NJ, USA; red arrow in Fig. 1(a)] attached to a phantom head was fixed to the headrest (height = 76 cm) of a typical dental chair using adhesive tape [Fig. 3(a)]. The nebulizer was then operated at 20 psi to generate stable aerosols using a sodium chloride solution as recommended by the U.S. National Institute of Occupational Safety and Health (NIOSH, TEB-APR-STP-0059).^56,57^ The spatial distribution of size-resolved (15 channels from 0.30 to 20.00 *μ*m) aerosol number concentration was measured (in triplicate, 70 different locations, 1.5 m apart, see Fig. 1) using two identical and previously calibrated portable real-time (time interval of 6 s) aerosol laser particle spectrometers (90° light scattering, λ = 780 nm, 40 mW; Model 1.108, GRIMM Aerosol Technik Ainring GmbH & Co. KG, Germany) with integrated 47-mm polytetrafluoroethylene (PTFE) filters that allows for fluorescent particles to be collected during optical measurements for further analyses (such as gravimetric and chemical analyses).

### C. Confocal laser scanning microscopy (CLSM)

Particles captured on the PTFE filters were further analyzed by confocal laser scanning microscopy (TCS SP8, Leica Microsystems, Germany) with three lasers (UV, violet, and white; λ = 405, λ = 442, and λ = 470 to 670 nm, respectively) and light-gating capabilities to reduce background noise. Images were collected in four different channels (red, green, yellow, and blue) at 600 Hz using a dry objective (10×, model = HC PL APO 0.4NA, Leica Microsystems, Germany) and two supersensitive spectral hybrid detectors. Histograms of confocal images acquired were generated using the ImageJ digital image analysis software (Version 1.53a, Wayne Rasband NIH, Bethesda, MA, USA; available online at http://imagej.nih.gov/ij/).^58^

### D. Operatory room

**FIG. 2.**
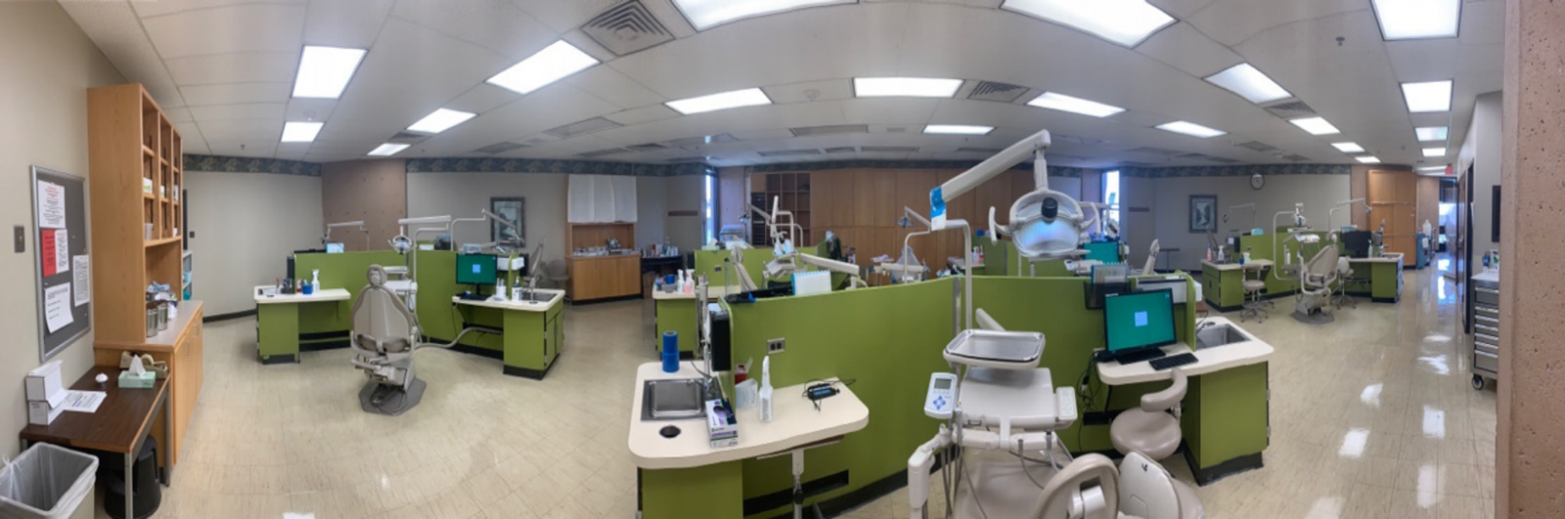
Green Clinic of the University of Oklahoma Health Sciences Center College of Dentistry.

Size-resolved spatial distribution measurements of aerosols generated using the portable Collison nebulizer took place in the Green Clinic (length = 19.2 m, width = 10.7 m, ceiling height = 2.7 m; area = 205.4 m^2^, volume = 554.7 m^3^, 25 °C, 55% relative humidity) in the University of Oklahoma Health Sciences Center College of Dentistry (OUCOD). The Green Clinic has 12 dental chairs (1.8 m apart) that are physically separated by wooden cabinets (1.2 m tall). The air handling system in the Green Clinic is capable of 6.5 air-exchanges-per-hour, which is in accordance with ANSI/ASHRAE standard 62.1 (2019).^59^ The rationale for the selection of the Green Clinic as the microenvironment for the present study was based on the fact that the air handling system installed has the lowest levels of air-exchanges-per-hour and the shortest cabinetry in the college (with are conditions known to favor aerosol distribution). The present study was conducted when the Green Clinic was empty to avoid unnecessary exposure of students, staff, and faculty members to the particles investigated. Therefore, the present study was not required to be reviewed or approved by the Institutional Review Board of the University of Oklahoma Health Sciences Center (Fig. 2).

### E. Aerosol containment device

**FIG. 3.**
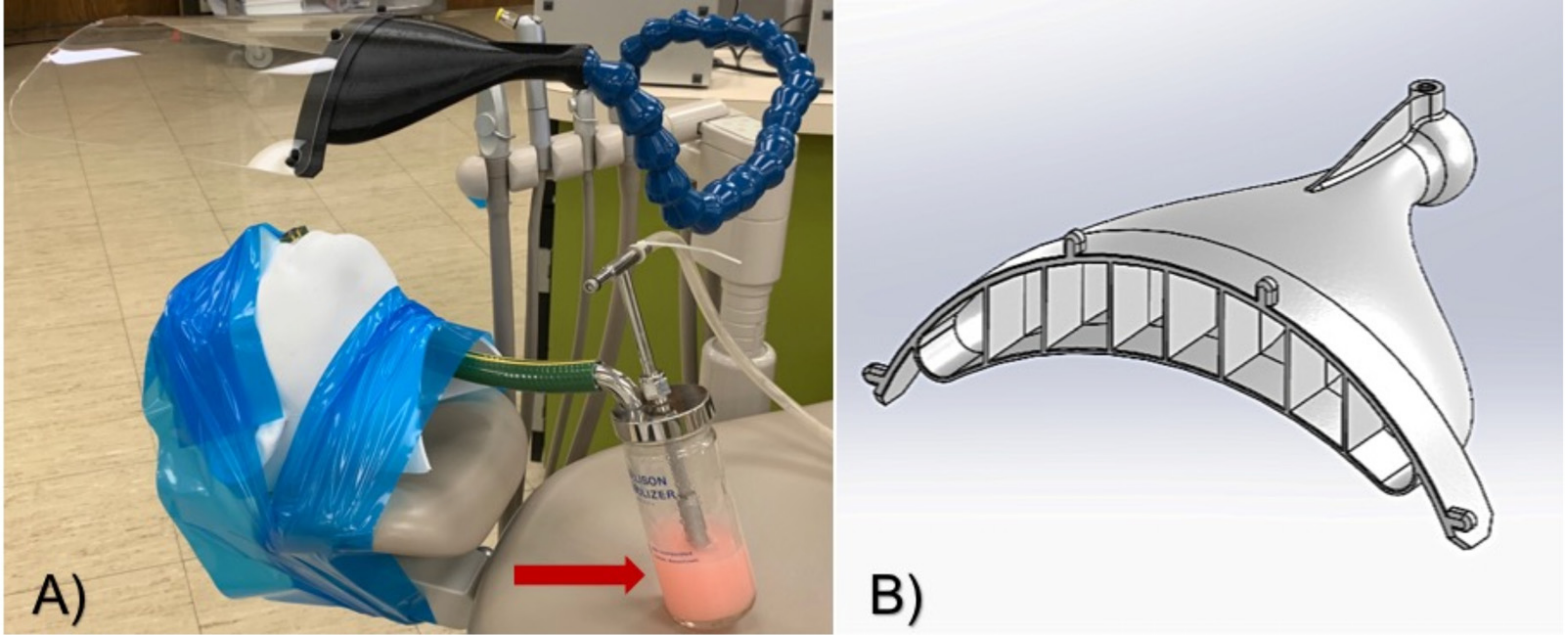
(a) Experimental setup displaying the phantom's head attached to the headrest of a dental chair (centrally located in the Green Clinic), the ACD positioned immediately above (13.00 cm) the phantom's head and the Collison nebulizer (red arrow) with fluorescent particles in 2% NaCl solution (pink colored). (b) Isometric view of the ACD.

A prototype aerosol containment device [ACD; Figs. 3(a) and 3(b)] was collaboratively developed by the University of Oklahoma Health Sciences Center College of Dentistry and the Tom Love Innovation HUB of the University of Oklahoma.

The ACD is composed of a plastic body and a transparent plastic shield. The body (length = 387.40 mm, width = 188.00 mm, height = 69.90 mm) was additively manufactured using polylactic acid (PLA) and a 3D printer (Craftbot Plus PRO, Craftbot, Budapest, Hungary; nozzle = 0.40 mm, layer height = 0.32 mm) with controlled bed and extruder temperatures (60 °C and 220 °C, respectively), whereas the transparent plastic shield (width = 238.12 mm, length = 200.00 mm) was laser cut and coupled to the body.

A commercially available Loc-line modular hose (internal diameter = 19.05 mm) with an integrated valve was then mounted to the ACD to allow it to be connected to most dental chairs in the United States and many models worldwide. The ACD was then connected to the high volume evacuation system (flow rate = 200 l/min) of one dental chair centrally located in the Green Clinic (see Fig. 1, blue chair) and was centrally aligned to the phantom's head (height = 13.00 cm) for the duration of size-resolved spatial measurements of aerosols generated, following the recommendations of a recent study.^2^

### F. Statistical analysis

Experimental data from the size-resolved spatial distribution measurements were statistically analyzed using General Linear Models (GLM) and Student–Newman–Keuls (SNK) post hoc tests (∝ = 0.05). Parameters of interest included the utilization of the ACD, the location where measurements took place (70 different locations, see Fig. 1) in the Green Clinic and the interaction between parameters of interest. Statistical analyses were performed using SAS software (Version 9.2; SAS Institute, Cary, NC, USA).

## III. RESULTS

**FIG. 4.**
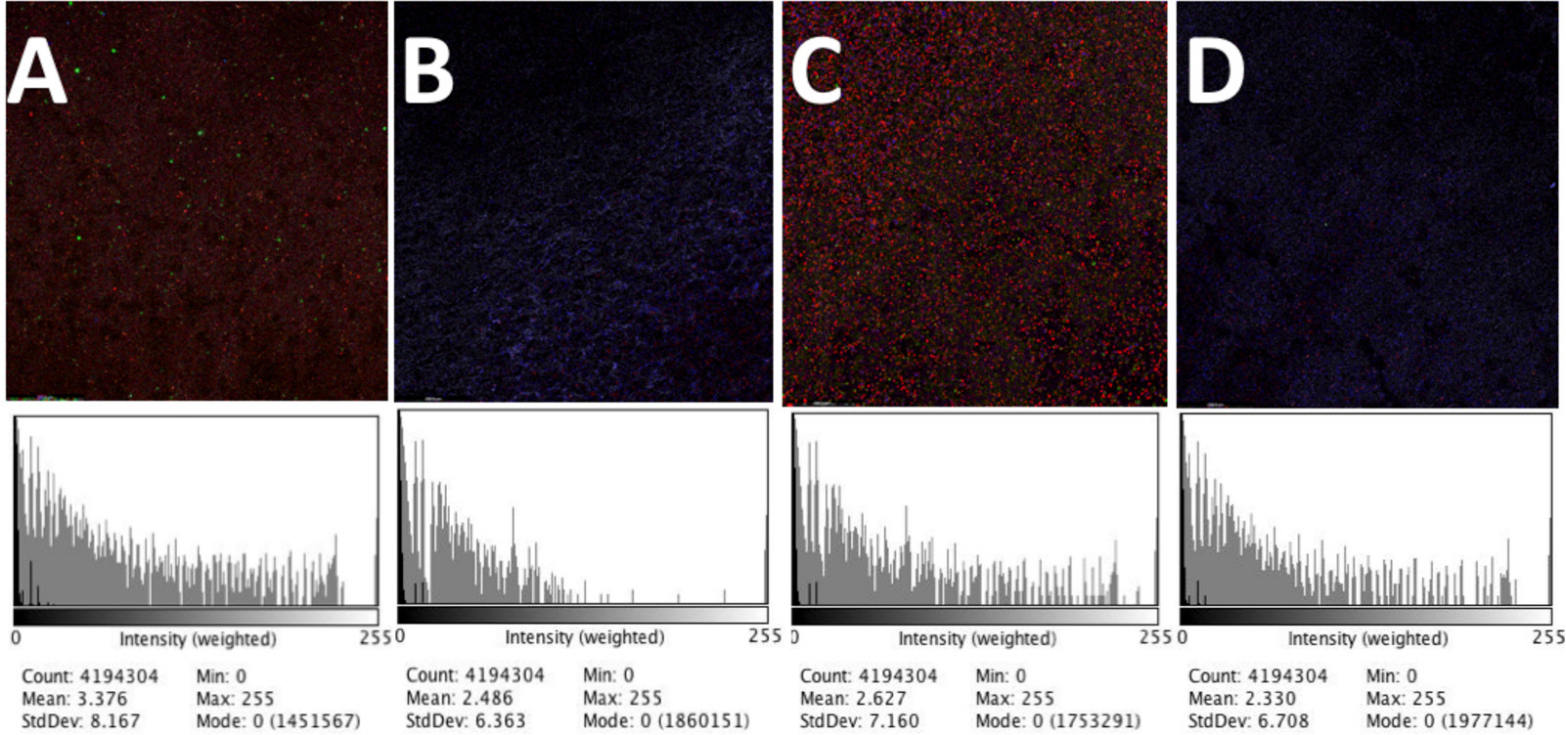
Confocal laser scanning microscopy images of fluorescent microspheres (0.25–20.00 *μ*m) captured on PTFE filters and their respective RGB-weighted histograms, demonstrating the numbers of particles collected varied from 3376 ± 8167 to 2627 ± 7160 [(a) and (c); no ACD] and from 2486 ± 6363 to 2330 ± 6708 [(b) and (d); with ACD]. Blue colors in (b) and (d) indicate the autofluorescence of PTFE filters and should not be confused with particles in (a) and (c).

Figures 4(a)–4(d) show representative CLSM images (magnification 10×) of fluorescent microspheres collected on PTFE filters used during the size-resolved spatial distribution analysis and Red/Green/Blue (RGB)-weighted histograms generated using ImageJ. CLSM images have demonstrated, in terms of mean fluorescence and standard deviation values, that number of particles collected on PTFE filters varied from 3376 ± 8167 to 2627 ± 7160 [Figs. 4(a) and 4(c), no ACD] and from 2486 ± 6363 to 2330 ± 6708 [Figs. 4(b) and 4(d), with ACD].

**FIG. 5.**
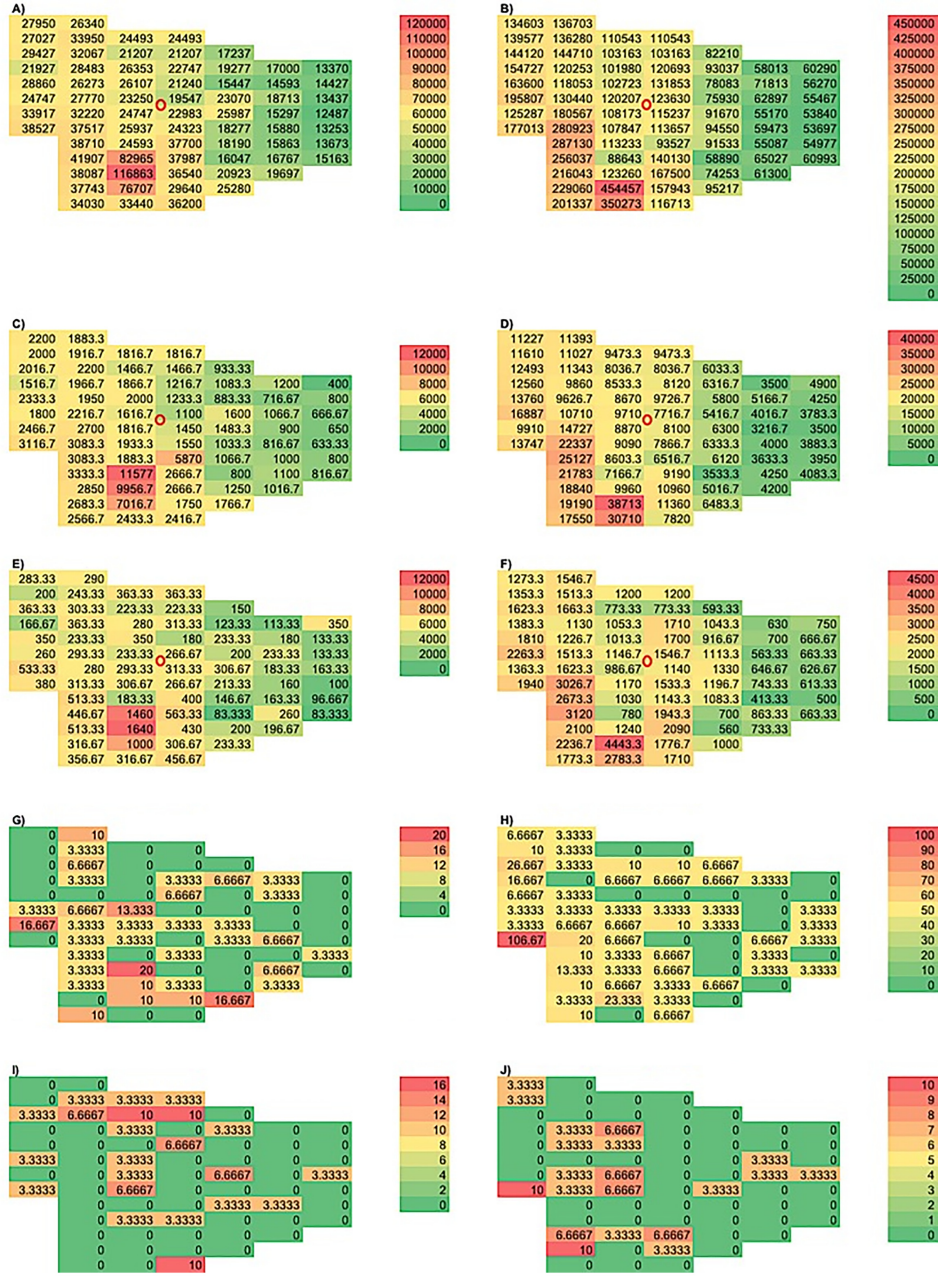
Size-resolved spatial distribution of particles with dimensions between (a) and (b) 0.25 and 0.50 *μ*m, (c) and (d) 0.50 and 1.00 *μ*m, (e) and (f) 1.00 and 3.00 *μ*m, (g) and (h) 4.00 and 5.00 *μ*m and (i) and (j) 5.00 and 20.00 *μ*m. Images shown illustrate particles' distribution behavior with (a), (c), (e), (g), (i) or without (b), (d), (f), (h), (j) the utilization of the ACD. The red circle in images (a)–(i) denotes the position of the Collison nebulizer.

**FIG. 6.**
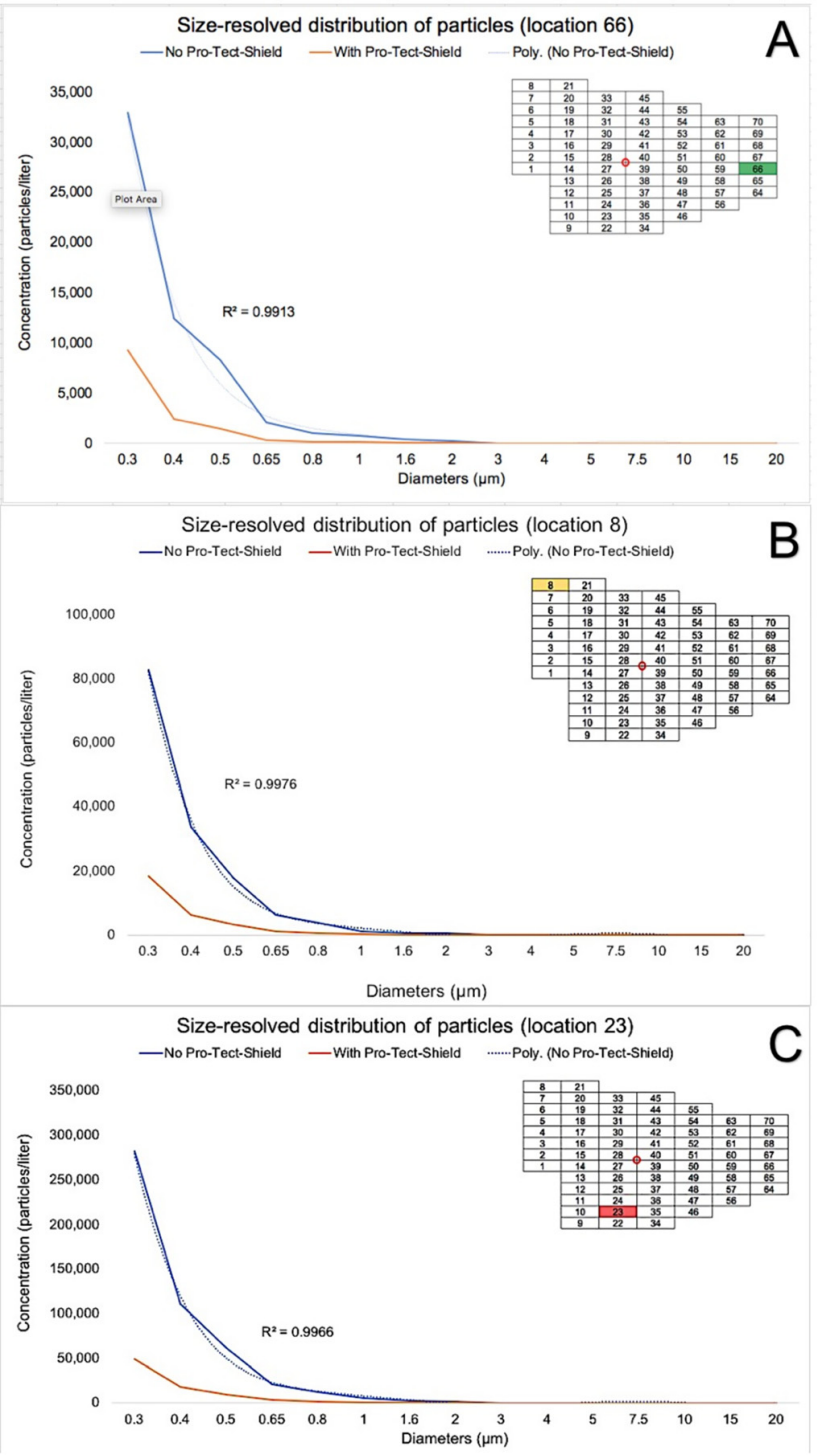
Size-resolved analysis of particles concentrations (in terms of particles/l) detected for locations (a) 66, (b) 8, and (c) 23 with (orange curves) or without (blue curves) the utilization of the ACD. These locations were selected because they had distinct particles' concentrations (low, intermediate, and high, respectively) and were localized approximately 6.0 m from the nebulizer. Insets in images (a)–(c) show the map of locations, the position of the collision nebulizer (red circle). Colors (orange, red, or green) in location maps indicate particles' concentrations as shown on (a)–(j).

The results from the size-resolved spatial distribution analysis performed are presented in Figs. 5 and 6 in terms of mean concentration values (particles/liter). Figures 5(a)–5(j) are formatted as color-coded maps to demonstrate the spatial distribution of particles (0.30–20.00 *μ*m) in the Green Clinic with (A, C, E, G, I) or without (B, D, F, H, J) the utilization of the ACD. Figures 6(a)–6(c) have been formatted as line graphs to emphasize the effect of particles' dimensions and engineering controls (ACD) on the spatial distribution of particles at three specific locations (66, 8, and 23) displaying particle concentrations that were significantly different (low, intermediate, and high, respectively) and were localized approximately 6.0 m from the nebulizer.

Figure 5(b) demonstrates that particles of size comparable to those of SARS-CoV-2 were successfully nebulized and efficiently spread throughout the area of the Green Clinic when the ACD was not used. Particles' concentrations detected varied from 53 697 ± 1673 to 454 457 ± 143 146 particles/l. Even though Fig. 5(a) shows a similar distribution behavior, the results reported have demonstrated that the utilization of the ACD has drastically reduced particles' concentrations detected independently of the location where measurements took place. Figures 5(c) and 5(d) show the distribution behavior for particles with dimensions between 0.50 and 1.00 *μ*m. It can be noticed that concentrations detected varied from 3500 ± 390 to 38 713 ± 12 171 particles/l [Fig. 5(d), no ACD] and from 566 ± 288 to 11 577 ± 8383 particles/l [Fig. 3(c), with ACD]. Figures 5(e) and 5(f) illustrate the distribution of particles with dimensions between 1.00 and 3.00 *μ*m, where it is possible to observe that mean concentration values varied from 500 ± 100 to 4443 ± 627 particles/l [Fig. 5(f), no ACD] and from 83.3 ± 76.37 to 1640 ± 741 particles/l [Fig. 5(e), with ACD]. Figures 5(g) and 5(h) display the spatial distribution results for particles with dimensions between 4.00 and 5.00 *μ*m. In this instance, concentrations identified varied between 0 and 107 ± 72 [Fig. 3(h), no ACD] and from 0 to 20 ± 17 particles/l [Fig. 3(g), with ACD]. Figures 5(i) and 5(j) show the results for particles with dimensions between 5.00  and 20.00 *μ*m where it can be observed that mean concentration values varied from 0 to 10 ± 10 particles/l [Fig. 5(i), no ACD] and from 0 to 10 ± 3.4 particles/l [Fig. 5(j), with ACD]. It is obvious from the results reported that the utilization of the ACD resulted in significant reductions in particles' distribution behaviors independently of the location considered (70 different locations), and its efficacy seemed to be more pronounced for particles of smaller sizes.

Figure 6 demonstrates particles (0.30–20.00 *μ*m) concentrations (in terms of particles/liter) detected for positions 66, 8, and 23, respectively. The results reported indicate that concentrations of particles distributed exponentially decreased in the function of increasing diameters and with the utilization of the ACD, which was demonstrated to promote significant reductions in particles concentrations [location 66 (by 3.72-fold), location 8 (by 4.43-fold), and location 23 (by 8.56-fold)]. It is clear that the utilization of the ACD drastically reduced the concentration of particles with dimensions that are similar to those previously shown^29^ to contain peak levels of SARS-CoV-2 (0.30–1.00 *μ*m) in two Chinese hospitals. It can also be observed that concentrations detected tended to zero with or without the utilization of the ACD when particles' diameters were above a specific threshold (∼3 *μ*m). Taken together, these results indicate that particles smaller than ∼3 *μ*m are generated far more abundantly than larger particles and that smaller particles (<3 *μ*m) are transported more efficiently than larger particles (>3 *μ*m). Based on the results reported, it was possible to rank order particles' distribution behavior as follows: 0.30–0.50 *μ*m  >  0.50–1.00 *μ*m  >  1.00–3.00 *μ*m  >  4.00–5.00 *μ*m  > 5.00–20.00 *μ*m.

## IV. DISCUSSION

The highly infectious nature of SARS-CoV-2 in combination with its airborne transmission routes (droplets and aerosols) and concerning mortality rates (2%–15%),^5^ resulted in a global event of extraordinary proportions characterized by the massive spreading of COVID-19 and hundreds of thousands of deaths.^60^ This worldwide public threat^2^ has shut down economies and overwhelmed the healthcare system of numerous countries, thereby imposing unprecedented occupational challenges for frontline healthcare workers (HCW) including nurses, physicians, and dentists. According to a previous systematic scoping review of the literature on bioaerosols, the risk of HCWs acquiring viral or bacterial infections directly correlates with the infectious nature of their patients, types of interventions and instruments used.^15^

In dentistry, handheld instruments (turbines, ultrasonic scalers, and air/water syringes) are considered the primary source of aerosols and splatter.^26,61,62^ A recent study^18^ investigating the topographical aspects of airborne contamination (on the dental chair and operatory room) caused by dental handpieces in clinical settings has demonstrated that contamination levels significantly varied based on location [dental chair (p <0.01) or operatory room (p < 0.0001)] and type of handpiece used [dental chair = air turbine (0.51 ± 0.17) > scaler (0.47 ± 0.14) > contra-angle (0.41 ± 0.14); operatory room = air turbine (0.26 ± 0.38) > contra-angle (0.20 ± 0.26) > 0.17 ± 0.27]. Findings have also indicated^18^ that contamination levels observed were high and fairly regular on the ceiling and walls of the operatory room, thereby suggesting an aerosol transmission mechanism and the need for disinfection protocols to include such surfaces.

The results of the size-resolved spatial distribution analysis reported in this study (Figs. 5 and 6) demonstrate that the novel ACD proposed reduced the airborne spreading of small particles of greatest concern for the transmission of COVID-19 in dental settings. The spreading of particles was observed to be distance-dependent and to inversely vary in function of particles' diameters (0.30 *μ*m > 0.40 *μ*m > 0.50 *μ*m > 0.65 *μ*m > 0.8 *μ*m > 1.00 *μ*m > 1.60 *μ*m > 2.00 *μ*m > 3.00 *μ*m > 4.00 *μ*m > 5.00 *μ*m > 7.50 *μ*m > 10.00 *μ*m > 15.00 *μ*m > 20.00 *μ*m). In addition, particles' concentrations were demonstrated to decay in a sixth-order polynomial manner [Figs. 6(a)–6(c), R^2^ values between 0.9914 and 0.9964], where concentrations detected were significantly (p < 0.0001) higher in locations adjacent (123 630 ± 6667 particles/l) to the nebulizer when compared to those located 6.0 m from the nebulizer (55 467 ± 1231 particles/l). The results of the present study have been corroborated by previous studies^18,26^ that have shown that dental bioaerosols may contaminate surfaces located between 45 and 360 cm from the bioaerosol source (dental handpieces).

Allison *et al.*^63^ while evaluating the aerosol and splatter generated following dental procedures (anterior crown preparation, full-mouth ultrasonic, and washing of premolar with air/water spray) using a fluorescein solution (2.65 mmol l^−^1), digital images, and a spectrofluorometric analysis have demonstrated the presence of strong fluorescein contamination levels (mannequin > operator > assistant) that decreased in the function of the distance from the aerosol source (2 > 4 m). However, despite the novelty associated with the utilization of fluorescein dyes to demonstrate the spatial distribution of aerosols in dental settings,^63^ the study cited has critical methodological limitations that include (i) the dissolution of fluorescein salts in de-ionized water (DIW) and (ii) the limited resolution of the CCD camera utilized. The first limitation precipitates from DIW's well-known ability to generate carbonic acid when exposed to atmospheric air, which not only significantly lowers DIW's pH to around 5.5, but also drastically decreases the fluorescence of fluorescein by 96.5%.^64^ The second limitation is related to the fact that the camera utilized (Canon EOS 1000D) has an approximate pixel pitch of ∼5.71 *μ*m (Ref. 65), which cannot resolve individual particles with sizes between 0.25 and 5.00 *μ*m. In combination, these limitations adversely impacted authors' ability to accurately describe fluorescein contamination levels that were visually perceivable at distances beyond 4 m from the aerosol source^63^ and could also have impacted the accuracy of the spatial distribution reported. These results are of strong relevance because Liu *et al.*^29^ while investigating the aerodynamics of SARS-CoV-2 in two Wuhan hospitals have recently demonstrated that peak viral concentrations were observed in particles between 0.25 and 0.50 *μ*m, and, therefore, further support the utilization of the methodology described in the present study.

The statistical analysis performed in the present study revealed significance (p < 0.0001) for both main effects [utilization of ACD (“cond”) and location (“pos”) with the Green Clinic] as well as significance for the interaction of the two main effects. This indicates that the ACD is capable of strong reductions in particle concentration at different distances from the source. Significant differences were not found (p > 0.05) for particles with dimensions between 0.50 and 20.00 *μ*m for parameters cond and pos. The results shown in Figs. 5(g)–5(j) have clearly indicated that particles of larger dimensions (4.00–20.00 *μ*m) were not capable to efficiently spread throughout the Green Clinic area, as denoted by values that ranged from 0 to 20 particles/l (with ACD) and from 0 to 106.67 particles/l (no ACD).

These findings have been corroborated by the study published by Vuorinen *et al.*,^6^ who indicated that large particles (>100 *μ*m) follow a ballistic trajectory and fall within a limited radius from the source, while intermediate (10 and 100 *μ*m)^66^ and small particles (<10 *μ*m)^66^ can float and spread much further under the influence of an airflow stream. Other studies^61,67–70^ have also supported the results of the present study and have demonstrated that the utilization of high-volume evacuation (HVE) systems is capable of reducing airborne contaminants in dental operatories by more than 90 percent. A recent study by Teichert-Filho *et al.*^71^ reported on a low-cost prototype device to reduce the spreading of aerosols in dental settings by physically isolating the patient in a rigid and transparent acrylic enclosure that is coupled to an air filtration system and an external chamber filled with 2% NaOCl solution for viral inactivation. Even though the prototype developed may contribute to the prevention of SARS-CoV-2 in dental settings, the prototype, as designed, has critical limitations that may decrease the widespread usability of the device, including patients that are claustrophobic, pediatric, or have special needs. Another critical limitation associated with the reported prototype relates to the time required to efficiently disinfect the prototype between patients and the disinfection of the aspiration system piping (deemed challenging by the authors).^71^ Another recent simulation study investigated the utility of a vacuum helmet to contain pathogen-bearing droplets in dental and otolaryngologic outpatient interventions.^72^ According to the results reported, the utilization of the simulated vacuum helmet could theoretically control 100% of airborne droplets and 99.6% of all cough droplets.^72^

The aerosol containment device reported in the present study was designed to be inexpensive, completely adjustable [vertical (y axis), horizontal (x axis), and diagonal (z axis)] and able to function in a hands-free manner so that the dentist and the dental assistant are not required to hold, guide, or aim the ACD to improve its aerosol removal efficiency.^10^ We propose that the novel ACD reported in the present study could potentially be widely used in Dental Schools and private practices across the United States in an attempt to meet the regulations proposed by the American Dental Association (ADA),^41^ the U.S. Centers for Disease Control and Prevention (CDC), and the U.S. Occupational Safety and Health Administration (OSHA) that “all procedures involving blood or other potentially infectious materials should be performed in such a manner as to minimize splashing, spraying, spattering, and generation of droplets of these substances.”^55^ We also recommend that follow-up studies be conducted to compare the efficacy of the ACD proposed in the present study to those of commercially available products previously reported.^73^

## V. CONCLUSIONS

The present study has successfully demonstrated that particles similar in size to those found in hospitals that have the highest concentration of SARS-CoV-2 (0.25–0.50 *μ*m) are capable of efficiently spreading throughout the area of a large operatory room in a dental school. A highly efficient, low-cost, and hands-free aerosol containment device was designed to meet OSHA regulations (29 CFR 1910.134) for the control of contaminated aerosols in dental settings. The utilization of the ACD was shown to significantly decrease particles' concentrations detected by up to 8.56-fold. The efficiency of the novel ACD proposed was shown to be higher for particles with dimensions between 0.25 and 0.50 *μ*m and, therefore, hold the promise of decreasing the potential for spreading of infectious respiratory diseases in dental settings.

## AUTHORS' CONTRIBUTIONS

F.L.E.F. conceptualized the study; conceptualized the ACD; produced maps, graphs, 2D models of the Green Clinic; and wrote the manuscript. T.T. was responsible for the CAD/CAM of the ACD and its final design, T.O. helped to conduct the experiment in the Green Clinic, and S.S.K. performed the statistical analysis and recommended minor design changes. E.F. conceptualized the study. C.C. conceptualized the study, conducted the study in the Green Clinic, and reduced data to produce figures. All authors have critically reviewed and approved the manuscript for publication.

## Data Availability

The data that support the findings of this study are available from the corresponding authors upon reasonable request.
